# A short review on pharmacological activity of Cissus quadrangularis

**DOI:** 10.6026/97320630016579

**Published:** 2020-08-31

**Authors:** Jeganath Sundaran, Raleena begum, Muthu Vasanthi, Manjalam Kamalapathy, Giridharan Bupesh, Uttamkumar Sahoo

**Affiliations:** 1 Department of Pharmaceutics, School of Pharmaceutical Sciences, Vels Institute of Science, Technology and Advanced Studies (VISTAS), Pallavaram, Chennai-600117, India; 2 Research and Development Wing, Central Research Laboratory, Sree Balaji Medical College and Hospital (SBMCH), BIHER, Chennai-600044, India; 3 Department of Forestry, Mizoram University, Aizawl, Mizoram -796004; 4 Department of Forest Science, Central University of Nagaland, Lumami, India

**Keywords:** Cissus quadrangularis L, pharmacology, medicinal plants, phyto-chemistry

## Abstract

Cissus quadrangularis L. is a succulent plant of family Vitaceae usually found in tropical and subtropical xeric wood. It is a beefy desert plant like liana generally utilized as typical nourishment in India. It finds application in medicine. Experts have made efforts to test the plant's suitability using rational analysis. Some of the pharmacological use of the plant are linked to cell reinforcement, free radical search, hostile to microbials, bone regeneration, ulceration, pain relief, mitigation and diuretics. Hence, we document the available pharmacological data on Cissus quadrangularis L in the literature for further use.

## Background

Cissus quadrangularisis is a perennial herb with medicinal properties distributed throughout the tropical world. It is one of the most frequently used medicinal plants in India. It is believed that the plant is native to India, Sri Lanka, Malaysia, Java and West Africa. This plant is studied for its phytochemical constitution, pharmacological activities and toxicological evaluation. It is used for bone healing [1-3]. Ayurveda prescribe this plant for several medicinal ailments. Cissus quadrangularis synonym Cissus succulent popularly known as horjora in Hindi and pirandai in Tamil belongs to the family Vitaceae. The plant is widely seen in tropical forest regions of Asia and Africa [4-6].

## Plant propagation:

It requires a warm tropical climate. It is propagated using the stem cutting methods in the months of June to July. The plant is efficiently reproduced using its mature stem cuttings. A disease free, healthy and mature plant of Cissus quadrangularis L. was used as a source of stem cuttings for further development. It can be directly grown in prepared beds with moderate supply of water and suitable substratum to climb. A 30 cm long mature stem was removed from their mother plant without damage for propagation.

**Figure 1:**
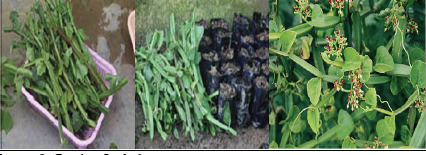
On day 0 of plant propagation

**Figure 2:**
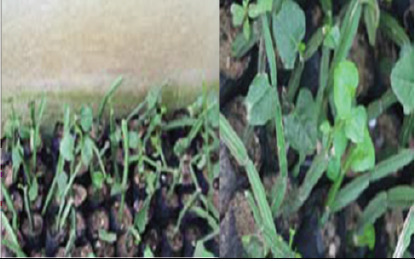
Variation in stem cutting after 20 Days

**Figure 3:**
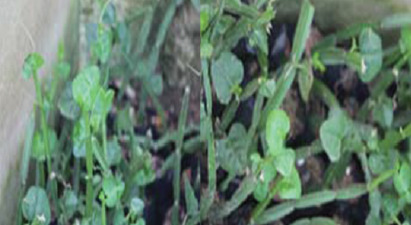
Variation in stem cutting after 30 Days

**Figure 4:**
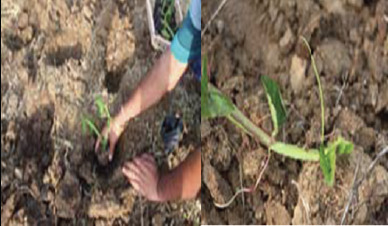
Transfer into the open field

One poly bag of size 13 cm length and 8 cm width filled with fertile soil, manure and sand equally which acts as a medium for its regeneration using their stems (10 cm deep in poly bag individually) in a Herbal Garden is used. Well-prepared soil, manure and sand mixture was made to support the growth of new shoots and roots. It is a succulent plant and excess water effect on the growth. After few days of stem cutting propagation new buds development starts and gradually the plant convert in to the field as a new plant like their mother plant (Figure 1 and Figure 2). A developed plant of Cissus quadrangularis L. is helpful to transfer the plants easily from one place to another as per need. Above activities not only support the plants for rapid multiplication but also for their dissemination (Figure 3 and Figure 4).

## Microscopic characters of the plant:

Transversely cut surface of young stem is rectangular in outline with discontinuous rings of vascular bundles. This is parallel to the under surface of the epidermis with 3 to 4 vascular bundles under the wings that is more developed than the ones at the flat sides. This is conjoint, collateral with a cap of bast fibres encircled by idio-blast containing cluster crystals of calcium oxalate, with numerous air cavities throughout the section. A complete ring of vascular strand with well-developed cambium ring is seen except at the flat broad side of the stem in old stem.

**Figure 5:**
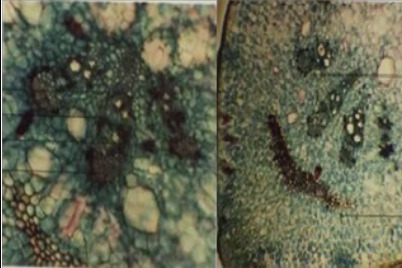
TS of stem showing one of the four corners

**Figure 6:**
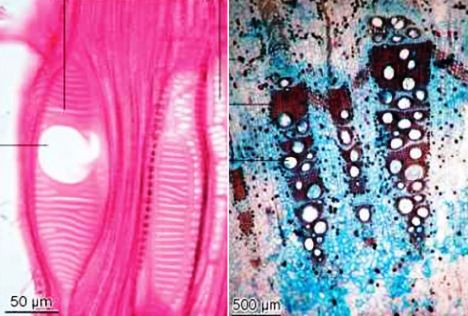
TS of stem showing the other one of the four corners

Actinocytic stomata transverse throughout the epidermis, which, in surface view, are seen, encircled by small cells forming a girdle like sheath. The epidermal cells are thick-walled and rectangular to pent angular in surface view. Cortex is composed of thin-walled parenchymatous cells containing chloroplasts, starch grains and rap hides of calcium oxalate. A colleen chymatous arc is present outside the vascular bundles in the cortex beneath each of the four angles (Figure 5 and Figure 6).

## Powder character of the plant:

The powder is creamish brown with a actinocytic stomata of stem. Clusters, rosettes, and crystals of calcium oxalate in bundles are scattered. Starch grains are mostly simple, vessels spiral, annular and pitted. Mucilage cavities encircled by a layer of epithelium fragments of pericyclic fibers associated with idioblast containing cluster crystals of calcium oxalate are seen [7,8].

## Chemical constituent of the plant:

The plant consists vof arious constituents (Table 1) such as flavanoides like quercetin, daidzein and genistein, triterpenoids like friedelin, vitamin 'C', stilbene derivatives like quadrangularin-A, resveratrol and piceatannol, iridoids like 6-0-meta-methoxy-benzozyl catapol, picroside and pallidol and phytosterols like β- sitosterol and calcium were identified as major constituents of the plant [9,10]. The stem parts of plant contains A and β-amyrins, β-sitosterol, ketosetosterol, phenols, tannins, vitamin, carotene, Calcium oxalate, 31 methyl tritiacontanoic acid, taraxeryl acetate, taraxeroliso-pentadecanoic acid, Calcium ions and phosphorus. The Aerial parts of the plant contain new asymmetric tetracyclic triterpenoid 7-Oxo-Onocer-8-ene-3 β 21-α diol. Leaves contain Resveratrol, piceatanon, pallidol, parthenocissus and alicyclic lipids. Root powder often provides a steady source of mineral resources including potassium 67.5 mg; calcium 39.5 mg, zinc 3.0 mg, sodium 22.5 mg, Iron 7.5 mg, lead 3.5 mg, cadmium 0.25 mg, copper 0.5 mg and magnesium; 1.15 mg [13-17].

## Utility of the plant:

Cissus quadrangularis is used for diabetes, obesity, high cholesterol, bone fractures, allergies, cancer, stomach upset, painful menstrual periods, asthma, malaria, wound healing, peptic ulcer disease, weak bones, weak bones (osteoporosis) and as body building supplements as an alternative to anabolic steroids.

[1] The herb is used for osteoarthritis, rheumatoid arthritis and osteoporosis.

[2] The roots and stems are used to treat fractures of the bones.

[3] The stem paste boiled in limewater is given for asthma.

[4] The herb powder is administered in treatment of hemorrhoids and certain bowl infections.

[5] Stem juice is used for scurvy, debilitating menstrual disorders, otorrhoea and epistaxis.

[6] The herb is fed to cattle to stimulate milk flow.

[7] The strong fleshy quadrangular stem is traditionally used to treat acid reflux of gastritis, eye disorders, piles and anemia.

## Pharmacological properties:

### Bone healing activity:

The anabolic steroid from the Cissus quadrangularis plant showed a marked influence in the rate of fracture healing by early generation of all connective tissue. Cissus quadrangularis contains vitamins and steroids, which are found to have specific effect on bone fracture healing [7].

### Anti obesity activity:

A study was performed using a Cissus quadrangularis formulation called cylaris. The study had a double blind, placebo-control design. Results showed test subject had decreased waist circumference body mass index reduced serum lipid levels [8].

### Anti-ulcerative activity:

The anti-ulcerative effect of Cissus quadrangularis extract on enzyme H+K+-ATPase that is deemed responsible for producing acidity in stomach is observed.

### Anti-diabetic activity:

Anti diabetic property of Cissus quadrangularis was noted in a study where dry powder of Cissus quadrangularis is obtained through ethyl acetate extraction. This is tested for diabetes induced in wister albino rats by administering alloxan [9,10].

### Antioxidant and free radical scavenging activity:

Methanol extract of Cissus quadrangularis exhibits strong antioxidant and free radical scavenging activity in in vitro and in vivo systems mainly due to the presence of β-carotene [11,12].

### Gastro protective Activity:

Because of its significant source of carotenoids, triterpenoids and ascorbic acid, Cissus quadrangularis is used for the gastrointestinal diseases in traditional medicine, and has gained significant recognition on human nutrition. Numerous studies demonstrated the impact of Cissus quadrangularis extract (CQE) on gastrointestinal toxicity and gastro-protective effect. This is together with its function underpinning the clinical intervention toward aspirin-induced gastric mucosal damage [13].

### Central nervous system activity:

The root extract possesses stimulant CNS function suggested by decreasing exploratory actions. Methanol root extract comprises saponins that exhibit powerful sedative action and also suppress spontaneous motor action in mice [14-16].

### Analgesic, anti-inflammatory and stimulatory activity:

Methanol extract has analgesic, non-inflammatory and venotonic impacts with hemorrhoids, non-inflammatory activity attributable to flavonoids and β-sitosterol. β-sitosterol in methanol extract does have the potential to reduce MPO enzymes. This indicate a significant decrease in the influx of neutrophils into the inflamed tissue. Ethanol extract has beneficial effect on neutrophils triggered by aspirin-induced tissue damage in rats [17].

## Conclusion

The phytochemical constituents and pharmacological action of the plant Cissus quadrangularis Linn is of significance. Thus, comprehensive information on Cissus quadrangularis is essential. Hence, we document the available pharmacological data on Cissus quadrangularis L in the literature for further use.

## Declaration on Publication Ethics:

The authors state that they adhere with COPE guidelines on publishing ethics as described elsewhere at https://publicationethics.org/. The authors also undertake that they are not associated with any other third party (governmental or non-governmental agencies) linking with any form of unethical issues connecting to this publication. The authors also declare that they are not withholding any information that is misleading to the publisher in regard to this article.

The authors are responsible for the content of this article. The Editorial and the publisher has taken reasonable steps to check the content of the article with reference to publishing ethics with adequate peer reviews deposited at PUBLONS.

## Figures and Tables

**Table 1:** Bioactive constituents of Cissus quadrangularis L

S. No.	Name of compounds
1	Alpha amyrin
2	Beta amyrin
3	Beta sitosterol
4	Friedelin
5	Quercetin
6	Genistein
7	Daidzein
